# Metabolic Syndrome and Inflammatory Responses to Long-Term Particulate Air Pollutants

**DOI:** 10.1289/ehp.10565

**Published:** 2008-02-25

**Authors:** Jiu-Chiuan Chen, Joel Schwartz

**Affiliations:** 1 Department of Epidemiology, University of North Carolina School of Public Health, Chapel Hill, North Carolina, USA; 2 Department of Environmental Health and; 3 Department of Epidemiology, Harvard School of Public Health, Boston, Massachusetts, USA

**Keywords:** air pollution, environmental health, metabolic syndrome, particles, risk factors, susceptibility, white blood cells

## Abstract

**Background:**

Human data linking inflammation with long-term particulate matter (PM) exposure are still lacking. Emerging evidence suggests that people with metabolic syndrome (MS) may be a more susceptible population.

**Objectives:**

Our goal was to examine potential inflammatory responses associated with long-term PM exposure and MS-dependent susceptibility.

**Methods:**

We conducted secondary analyses of white blood cell (WBC) count and MS data from The Third National Health and Nutrition Examination Survey and PM_10_ (PM with aerodynamic diameter < 10 μm) data from the U.S. Environmental Protection Agency Aerometric Information Retrieval System. Estimated 1-year PM_10_ exposures were aggregated at the centroid of each residential census-block group, using distance-weighted averages from all monitors in the residing and adjoining counties. We restricted our analyses to adults (20–89 years of age) with normal WBC (4,000–11,000 × 10^6^/L), no existing cardiovascular disease, complete PM_10_ and MS data, and living in current residences > 1 year (*n* = 2,978; age 48.5 ± 17.8 years). Mixed-effects models were constructed to account for autocorrelation and potential confounders.

**Results:**

After adjustment for demographics, socioeconomic factors, lifestyles, residential characteristics, and MS, we observed a statistically significant association between WBC count and estimated local PM_10_ levels (*p* = 0.035). Participants from the least polluted areas (1-year PM_10_ < 1st quartile cutoff: 27.8 μg/m^3^) had lower WBC counts than the others (difference = 145 × 10^6^/L; 95% confidence interval, 10–281). We also noted a graded association between PM_10_ and WBC across subpopulations with increasing MS components, with 91 × 10^6^/L difference in WBC for those with no MS versus 214, 338, and 461 × 10^6^/L for those with 3, 4, and 5 metabolic abnormalities (trend-test *p* = 0.15).

**Conclusions:**

Our study revealed a positive association between long-term PM exposure and hematological markers of inflammation and supported the hypothesized MS-dependent susceptibility.

Ambient air pollution, especially particulate matter (PM), has increasingly been recognized as a threat to cardiovascular health (Kaiser 2005; Nel 2005). As noted in a recent comprehensive review of the mounting volume of epidemiology literature (Pope and Dockery 2006), multicity air pollution studies using time-series or case–crossover analyses have reported fairly consistent associations between elevated PM concentrations over short periods of one or several days and increased cardiovascular mortality and morbidity (e.g., hospitalizations, emergency visits). Compelling data also link PM directly to specific cardiac events, including life-threatening arrhythmia (Dockery et al. 2005; Peters et al. 2000) and myocardial infarction (Zanobetti and Schwartz 2005). Since the 1990s, convincing evidence from large cohort studies has consistently demonstrated that long-term exposures to particulate air pollutants are associated with increased mortality. Both the Harvard Six-Cities Study (Dockery et al. 1993) and the American Cancer Society cohort study (Pope et al. 1995) reported that people residing in more polluted areas with high PM_2.5_ (PM with aerodynamic diameter < 2.5 μm), compared with those in less polluted areas, were more likely to die, and stronger associations were found with cardiopulmonary deaths. Yearly average of PM_10_ (aerodynamic diameter < 10 μm) was also associated with increased risks for hospitalization for congestive heart failure or recurrent heart attack among patients with previous myocardial infarction (Zanobetti and Schwartz 2007).

More recently, environmental health scientists have begun to elucidate the pathophysiologic mechanisms underlying the observed adverse short-term and long-term cardiac effects of ambient air pollution, and several interrelated mechanistic pathways have been proposed. Because of the pivotal role of vascular inflammation in pathogenesis and progression of atherosclerosis and coronary heart diseases, systemic inflammatory response to inhaled ambient particles has emerged as an important mediator of PM-associated acute cardiac effects (Pope 2000; Utell et al. 2002). Although much progress has been made in understanding the mechanisms of acute cardiac effects, human data are scant and conflicting with respect to the pathophysiologic mediators of cardiovascular disease (CVD) associated with long-term PM exposure. For instance, short-term elevation of ambient PM is associated with increased levels of inflammatory markers such as white blood cell (WBC) count (Liao et al. 2005; Schwartz 2001) and C-reactive protein (Peters et al. 2001; Pope et al. 2004). However, the link between inflammation and long-term PM exposure in human is still lacking.

The increasing prevalence and large number of U.S. adults (with an age-adjusted prevalence of 23–24%) with metabolic syndrome (MS) has imposed a major public health concern and presented a great challenge to health care (Ford et al. 2002; Grundy et al. 2004). A known precursor of CVD and type 2 diabetes (Wilson et al. 2005), the MS comprises a cluster of abnormalities that occur as a result of perturbations in multiple metabolic pathways, leading to central adiposity, insulin resistance, hyperglycemia, atherogenic dyslipidemia, and hypertension. Recent epidemiologic data have suggested that the presence of certain component abnormalities of MS, such as obesity (Chen et al. 2007; Dubowsky et al. 2006; Schwartz et al. 2005), hypertension (Holguin et al. 2003; Liao et al. 2005; Park et al. 2005), and diabetes mellitus (O’Neill et al. 2005; Park et al. 2005), may impart greater susceptibility to PM-associated cardiac effects. A lack of understanding of who is most at risk or susceptible is one of the most important gaps in our current knowledge regarding PM-related health effects (Pope and Dockery 2006). However, no prior studies have examined MS-dependent inflammatory responses to long-term PM exposure.

The following secondary data analyses were carried out to address these significant data gaps. We hypothesized that long-term PM exposure is associated with increased systemic inflammation, and that people with MS have a higher degree of inflammatory responses to PM.

## Materials and Methods

### Health data source

The extant health data came from the Third National Health and Nutrition Examination Survey (NHANES III) conducted by the National Center for Health Statistics of the Centers for Disease Control and Prevention between 1988 and 1994. Details about this survey and related methods have been published (National Center for Health Statistics 1995). In brief, the NHANES III followed a multistage stratified random sampling of the U.S. population, with oversampling of minorities (African Americans, Mexican Americans) and the elderly (≥ 60 years of age). The survey consisted of an extensive household interview followed by a series of laboratory and other physical tests in a mobile examination center (MEC). Only those who completed the household interview were invited for the MEC examination. NHANES III was conducted in two phases, 1989–1991 and 1991–1994, each sampling approximately 20,000 subjects from 44 and 45 communities, respectively, that comprised a national probability sample. The current study was restricted to health data collected in 1989–1991, because we accessed only geocodes of phase 1 participants.

### Study population of current analyses

To parallel previous analyses on MS in U.S. adults (Ford et al. 2002; Ninomiya et al. 2004), our study focused on NHANES III participants 20–89 years of age, although the entire survey covered all civilian, noninstitutionalized persons ≥ 2 months of age. Other eligibility criteria included *a*) no existing or prior histories of heart attack or stroke as told by physicians; *b*) living in current residence > 1 year; *c*) WBC count between 4,000 and 11,000 cells × 10^6^; and *d*) having complete laboratory and physical data to define the presence or absence of MS. We excluded all subjects with WBC > 11,000 or < 4,000 cells × 10^6^/L because they likely reflected either acute pathologic processes or underlying diseases.

### Outcome data

For the current analysis, we used the between-individual difference in WBC count as a marker of systemic inflammation. WBC count was included as part of the complete blood count performed using the Coulter Counter Model S-PLUS JR automated hematology analyzer (Coulter Electronics, Hialeah, FL). WBC count is an inflammatory marker and is considered a potentially useful predictor of prevalent or incident CVD, according to the joint scientific statement by the Centers for Disease Control and Prevention and the American Heart Association (Pearson et al. 2003). Although C-reactive protein is also a valid inflammatory biomarker for CVD, the NHANES laboratory tests did not use a high-sensitivity assay for C-reactive protein until 1999–2000.

### Ambient air pollution data

During the period of NHANES III, the only environmental monitoring data of ambient particles available nationwide were for PM_10_. We obtained the PM_10_ data from the U.S. Environmental Protection Agency (EPA) Aerometric Information Retrieval System (AIRS), which has evolved into the current U.S. EPA Air Quality System and contains information on all routine air pollution monitoring (U.S. EPA 2007). The approach to estimating daily exposure levels of major criteria air pollutants (PM_10_, ozone, nitrogen dioxide, sulfur dioxide) has been published elsewhere (Schwartz 2001). In brief, air pollution exposure was assigned to subjects on the basis of geocoding. The residential information needed for geocoding was obtained through an agreement with the National Center for Health Statistics. Each participant in NHANES III was assigned the latitude and longitude of the population centroid of the census block group in which they lived. Block groups are collections of adjoining blocks with populations of 500–1,000 persons. The boundaries of block groups are chosen to maximize similarity within group on socioeconomic factors. The latitude and longitude of each monitor was also retrieved from AIRS (U.S. EPA 2007). Individuals were assigned exposure values equal to the weighted average of all monitors in their county of residence and adjoining counties, with weights proportional to the inverse of the square of the distance between their residence and the monitor. The use of all monitors in the county of residence and adjoining counties allowed geographic variability in exposure within area to be reflected in the exposure measures.

### Definition of MS

The profiling of MS characteristics required data from the NHANES III adult questionnaire, MEC examination, and laboratory tests. We applied condition-specific cut points for MS, using the criteria given by the National Cholesterol Education Program (NCEP) for Detection, Evaluation, and Treatment of High Blood Cholesterol in Adult Treatment Panel III (NCEP 2001), with similar minor modifications as used by others (Ford et al. 2002; Ninomiya et al. 2004). The five component conditions are insulin resistance (IR), high blood pressure, hypertriglyceridemia, low high-density lipoprotein cholesterol (HDL-C), and abdominal obesity. IR was defined as fasting glucose ≥ 110 mg/dL; for this analysis, IR could also be defined by a self-report of current use of insulin or oral hypoglycemics. High blood pressure was defined as systolic blood pressure ≥ 130 mmHg or diastolic blood pressure ≥ 85 mmHg. For this analysis, high blood pressure could also be defined by a self-report of current use of antihypertensive medication. Hypertriglyceridemia was identified based on triglycerides ≥ 150 mg/dL. Low HDL-C was identified by HDL-C < 40 mg/dL in men or < 50 mg/dL in women. Abdominal obesity was defined as a waist circumference > 102 cm in men or > 88 cm in women.

We measured fasting plasma glucose using a modified hexokinase enzymatic method (Cobas Mira assay; Roche, Basel, Switzerland). The interassay coefficient of variation was < 4% during the entire 6 years of survey. We evaluated history of diabetes based on a positive response to either of the questions “Are you now taking insulin?” or “Are you now taking diabetes pills to lower your blood sugar? These are sometimes called oral agents or oral hypoglycemic agents.” Blood pressure was measured by a board-eligible physician. Treatment for hypertension was identified by positive responses to all three of the following questions: “Have you ever been told by a doctor or other health professional that you had hypertension, also called high blood pressure?”; “Because of your high blood pressure/hypertension, have you ever been told by a doctor or other health professional to take prescribed medicine?”; and “Are you now taking prescribed medicine?” Triglycerides and HDL-C were measured using a Hitachi 704 Analyzer (Boehringer Mannheim Diagnostics, Indianapolis, IN). Waist circumference was measured, by a trained examiner, at the midpoint between the bottom of the rib cage and above the top of the iliac crest from participants at minimal respiration to the nearest 0.1 cm.

### Relevant covariate data

Information on relevant covariates was collected through a structured interview. We determined the following covariates as important confounders: age, sex, race/ethnicity (non-Hispanic white, non-Hispanic black, Hispanic, and other), socioeconomic status [education (< 8, 8–12, > 12 years), annual family income (< $20,000, $20,000–35,000, $35,000–50,000, > $50,000), employment status], family size, poverty–income ratio (in quartiles), and degree of urbanization of the residential neighborhoods. We used these latter two variables to measure some contextual characteristics other than the ambient air pollution exposure within the residential environment. The poverty–income ratio, as derived by comparing the self-reported family income to the U.S. Census–based poverty threshold value for each calendar year adjusted for inflation and the age of the family reference person, was a relative poverty index with its values < 1.00, indicating that the family is considered poor relative to the official poverty threshold (National Center for Health Statistics 1995).

The degree of urbanization was based on the U.S. Department of Agriculture Rural–Urban codes and classified into two categories (urban, rural) (Butler and Beale 1994). All these factors are likely to determine where the participants live (and thus the estimated PM_10_ exposure level), and they are also known to predict CVD. Smoking status (never, past, current smoker) and frequency of alcohol consumption were considered potential confounders. We also extracted the following data to be included in the sensitivity analyses: potential sources of indoor air pollutants (environmental tobacco smoke, wood stove, fireplace, gas stove) and exercise activities (jogging/running, aerobic exercise). Substantial exposure to airborne particles from indoor sources may correlate with outdoor PM exposure, thus raising the concern of confounding. Physical activities may determine the level of exposure to ambient air pollutants, and the frequency of engaging in jogging/running and aerobic exercise was associated with inflammatory markers in NHANES III participants (King et al. 2003).

### Statistical analyses

Because NHANES participants were sampled from communities, WBC counts of individuals from the same cluster were expected to correlate with each other, likely because of geographic difference in population composition, nutritional status, and characteristics of residential neighborhoods (e.g., built environment, meteorologic factors). To account for this spatial autocorrelation, we used mixed-effects models to estimate the association between WBC count and estimated community-level exposure to PM_10_. The constructed mixed-effects models allow for regression analyses with a covariance structure assuming a common correlation within these sampling units and a random-effects term for each community, providing proper standard error estimates for the regression parameters under these circumstances. We compared the average WBC count across communities defined by the quartile distribution of PM_10_ 1 year before the examination. The potential modification of PM effect by the presence of MS was examined by comparing the spatial difference in WBC count, as putatively related to 1-year PM_10_ levels, across subgroups defined by the number of MS component abnormalities. The final mixed-effects models included the number of MS component abnormalities and also adjusted for age, sex, race, socioeconomic factors (education, income, employment status, poverty–income ratio, family size), smoking status, alcohol consumption, and urban–rural difference. All these analyses were restricted to NHANES III phase 1 participants who had available geocoded information with estimable PM_10_ data and fulfilled the afore-mentioned eligibility criteria. As a result, we noted that none of the assigned sampling weights in the publicly accessible data files are appropriate to conduct weighted regression analyses. We also carried out additional analyses to examine the possible confounding by concurrent exposure to indoor air pollutants or by exercise activities and to evaluate whether our findings on effect modification by MS were sensitive to the exclusion of subjects with known hypertension and diabetes mellitus before the MEC examination. All mixed-effects models were constructed using STATA 8.1 software (StataCorp., College Station, TX) with the xtreg command.

## Results

### Characteristics of study population and ambient PM_10_ exposure

Among those 44 communities selected in phase 1 of NHANES III, 33 had PM_10_ monitoring stations operating within the residing counties or adjoining counties in 1988–1991. Table 1 shows the demographic characteristics of U.S. adults participating in phase 1 of NHANES III. Compared with participants with estimable PM_10_ (*n* =5,369), subjects with missing exposure data were slightly older (*p* = 0.09), more likely to be minorities (*p* < 0.0001), and had higher MS prevalence (*p* = 0.002) and WBC count (*p* < 0.0001). These comparisons denoted that the missing data structure of PM_10_ exposure levels was dependent on demographic and geographic differences, making extant NHANES III sampling weights not applicable to our analyses. There were 2,978 adults (48.5 ± 17.8 years of age) who had estimable 1-year PM_10_ and met the eligibility criteria (no prior heart attack or stroke, normal WBC count, living in current residences > 1 year, complete MS profiles). As a result of these restrictions, there were more Hispanics but fewer non-Hispanic blacks (*p* < 0.0001) in our study, and they had lower WBC counts than those excluded subjects with estimable PM_10_, although age, sex distribution, and MS prevalence were comparable. Our study population had fewer active smokers (*p* = 0.004), and they were more likely to be of high socioeconomic status (*p* < 0.0001 for comparison of family income and poverty–income ratio) and live in urban areas (*p* = 0.008). However, there was no difference (*p* = 0.47) in the exposure distribution between our study population (1-year average PM_10_ ± SD: 36.8 ± 13.0 μg/m^3^) and those excluded from the analyses (37.5 ± 13.1 μg/m^3^). We also noted that 94% of the observed variability in 1-year average PM_10_ could be attributable to between-community difference.

The population correlates across communities defined by the quartile distribution of 1-year PM_10_ are presented in Table 2. As expected, older participants (*p* < 0.0001) and those with higher socioeconomic positions (*p* < 0.0001 for both education and income comparisons) were more likely to reside in the clean air communities, and only 53% of such communities were located in urban areas (vs. 71–77% for the other polluted communities; *p* < 0.0001). In the most polluted communities, there were more minority populations (Hispanics and others: 68% vs. 21%, compared with the clean air communities), and the majority (78%) were located in urban areas. Interestingly, the most polluted communities also had the highest MS prevalence compared with the others (28% vs. 21–26%; *p* = 0.045)

### Associations between PM_10_ and WBC count

In Table 3, we tabulated the statistically significant difference in geographic distribution of average WBC count across communities defined by the quartile distribution of PM_10_ 1 year before the examination (*p* = 0.01). Subjects from the clean air communities (in the 1st quartile of 1-year PM_10_) had the lowest WBC count (6,745 ± 81 × 10^6^/L), whereas the highest average WBC counts (7,094 ± 60 × 10^6^/L) were found in the most polluted communities (in the 4th quartile of 1-year PM_10_). We present in Table 4 the results of multivariable-adjusted mixed-effects models to estimate the effect of PM_10_ exposure on WBC count, by comparing subjects from the clean air communities (all with estimated 1-year PM_10_ < 1st quartile cutoff: 27.8 μg/m^3^) with all others residing in more polluted areas. In the crude analysis, this spatial difference in average WBC count associated with PM_10_ exposure was 239 × 10^6^/L [95% confidence interval (CI), 58–420]. This effect estimate was diminished to 145 × 10^6^/L (95% CI, 10–281) but remained statistically significant (*p* = 0.035) after adjustment for age, sex, race, socioeconomic factors (education, household income, employment status, poverty–income ratio, family size), smoking status, alcohol consumption, urban–rural difference, and the number of MS component abnormalities (model 1 of Table 4). As expected, the presence of MS is consistently associated with significant systemic inflammation (*p* < 0.0001), with WBC count increased by 204 × 10^6^/L (95% CI, 156–254) for each MS component abnormality.

### Effect modification by the degree of MS

**Figure 1:**
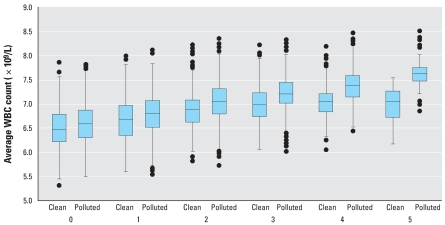
Differential inflammatory responses to long-term PM exposure according to the degree of MS. A graded increment of spatial difference in average WBC count compares participants residing in clean air communities (1st quartile of 1-year PM_10_) with those living in the other, more polluted areas associated with increasing number of MS component abnormalities (*p* = 0.15 for the trend-test; *p* = 0.044 after excluding subjects with established hypertension and/or diabetes mellitus).

Figure 1 depicts the MS-dependent spatial difference in average WBC count associated with PM_10_ exposure. Among those without MS, there was no appreciable spatial difference in average WBC count comparing subjects from the clean air communities with others residing in more polluted areas. The corresponding mixed-effects model revealed a graded association between PM_10_ and WBC count as the number of MS components increased, with 91 × 10^6^/L difference in WBC estimated for those with no MS versus 214, 338, and 461 × 10^6^/L for those with three, four, and five metabolic abnormalities, respectively (trend-test *p* = 0.15).

### Sensitivity analyses

Results of our sensitivity analyses (model 2 and model 3 of Table 3) conformed to the previous notion of very little confounding of PM effect on cardiovascular risks by indoor sources of air pollution or other lifestyle behaviors in the NHANES III participants (Schwartz 2001). After we added potential sources of indoor air pollutants and subsequently exercise activities to the adjusted mixed-effects models, we found only minor changes to the effect estimate (with spatial difference in WBC count: 141 × 10^6^/L, *p* = 0.041 and 138 × 10^6^/L, *p* = 0.046, respectively). Results of our sensitivity analysis also support the finding on MS-dependent spatial difference in average WBC count associated with PM_10_ exposure. After excluding those subjects (*n* = 700) with existing physician-diagnosed hypertension or diabetic mellitus and updating the corresponding mixed-effects model, we still observed a graded association between PM_10_ and WBC count, showing an even greater difference in WBC count as the number of MS abnormalities increased (with 87 × 10^6^/L difference in WBC count estimated for those with no MS, versus 353, 617, and 886 × 10^6^/L for those with three, four, and five metabolic abnormalities), and the test of effect modification by MS status became statistically significant (trend-test *p* = 0.044).

## Discussion

Our study results demonstrated that hematologic inflammatory markers of increased CVD risks, as reflected by increased WBC count, were associated with long-term (1 year) PM_10_ exposure, and such an association could not be attributable completely to the between-individual differences in demographics, socioeconomic factors, lifestyles, urban–rural locations, sources of indoor air pollutants, and conventional CVD risk factors used to define MS. This study provided the first epidemiologic data linking inflammatory biomarkers to long-term PM exposure and supports the hypothesized involvement of inflammation in PM-mediated chronic cardiovascular effects. Previously, large population-based cohort studies, such as the Framingham Heart Study (Kannel et al. 1992) and the Atherosclerosis Risk in Communities Study (Lee et al. 2001), reported an increase in long-term CVD risks associated with high WBC count. More recent data from the Women’s Health Initiative Observational Study further demonstrated WBC count as a stable and well-standardized marker of vascular inflammation independent of C-reactive protein in predicting long-term CVD events (Margolis et al. 2005). Short-term elevations of ambient PM_10_ levels were also associated with increases in WBC count (Liao et al. 2005; Schwartz 2001). Taken together, these scientific data highlight the importance of inflammation in the mechanistic pathways underlying both acute and chronic adverse health effects of PM exposure.

The observed differential inflammatory responses across individuals with different degrees of metabolic abnormalities support the concept that the presence of MS may impart greater susceptibility to PM-associated long-term cardiovascular effects. This new evidence is in line with previous epidemiologic observations that individuals with obesity (Chen et al. 2007; Dubowsky et al. 2006; Schwartz et al. 2005), hypertension (Holguin et al. 2003; Liao et al. 2005; Park et al. 2005), and diabetes mellitus (O’Neill et al. 2005; Park et al. 2005) are more susceptible to PM-associated acute cardiac effects. It also conforms to recent data from the Women’s Health Initiative Observational Study, where investigators found a graded increase in the relative risk for incident CVD associated with long-term ambient PM_2.5_ exposures among postmenopausal women with increasing level of obesity (Miller et al. 2007).

The positive association we found between long-term PM exposure with increased inflammatory responses as well as the empirical evidence on the enhanced susceptibility among subjects with MS is also consistent with a small number of toxicologic studies that just began to elucidate the mechanisms of chronic PM effects on CVD, using chronic exposures in animals with underlying metabolic abnormalities. In a susceptible animal model of Watanabe heritable hyperlipidemic rabbits (Suwa et al. 2002), instillation with PM_10_ (collected from outdoor air in Ottawa, Ontario, Canada) for 4 weeks was found to accelerate the progression of coronary atherosclerosis. PM_10_-instillation also increased plaque cell turnover and extracellular lipid pools in both coronary and aortic atherosclerotic lesions. In one study of concentrated air particles (Sun et al. 2005), after exposing apolipoprotein E-null mice to PM_2.5_ for 6 months (with equivalent concentration of 15.2 μg/m^3^, collected in Tuxedo, NY), researchers found that transverse sections of abdominal aorta increased 1.58-fold in percentage plaque area among mice maintained on high-fat, but not regular, chow. Further morphometric and immunohistochemical analyses indicated that vascular inflammation was elevated in atherosclerotic plaques of PM-exposed mice.

Our study findings raise several mechanistic questions that remain to be answered. For instance, what are the toxicokinetic and/or toxicodynamic features underscoring the PM-induced long-term inflammation among individuals with MS that can explain the observed MS-dependent inflammatory responses? In an inhalation study of healthy children 6–13 years of age, body mass index was associated with a graded increase in the estimated total lung dose of deposited fine particle (i.e., deposited particles/time) (Bennett and Zeman 2004), although whether obesity also increases particle deposition in adults with MS has not been demonstrated. Does any existing systemic inflammation, such as that reflected by increased WBC count in persons with MS, provide some mechanistic substrates predisposing affected individuals to an increased inflammatory response to airborne particles? This speculation, however, is not supported by empirical results of our sensitivity analyses, where we found an even greater difference in WBC count associated with 1-year PM_10_ after excluding those who had physician-diagnosed hypertension or diabetes mellitus, two clinical conditions with presumably high degrees of systemic inflammation. Interestingly, recent analyses of the Women’s Health Initiative Observational Study cohort data showed that women with prior hypertension or diabetes did not have heightened risks for CVD-associated long-term PM_2.5_ exposure when compared with women with no hypertension or diabetes (Miller et al. 2007). These data may suggest a complex pattern of interaction in human studies when the indicators of individual susceptibility involve clinical comorbidities. For instance, the concurrent use of medications such as anti-inflammatory agents may impart different levels of susceptibility not captured in our analyses. It is also arguable that residential mobility may depend on clinical comorbidities, making 1-year average not a good surrogate of long-term exposure among those with preexisting hypertension or diabetes. Biologically speaking, these findings may imply that future research should look into some preclinical pathophysiologic processes that likely parallel either the toxico-kinetic or toxicodynamic pathways underlying the MS-dependent long-term effect of PM exposure.

We recognize that our secondary data analysis has the following limitations that call for future studies to fully address them: First, because of its cross-sectional design, the observed association between increased WBC count and estimated 1-year PM_10_ exposure before the laboratory examination cannot be interpreted as definitely causal without any caution. Although we could not provide any good reasons why individuals with preexisting normal but relatively low WBC counts might have preferred or chosen to live in clear air communities, it is statistically arguable that residents from clean air communities might happen to have WBC counts lower than others at baseline. Second, although we have adjusted for a large set of individual-level confounders and some other residential characteristics, we could not rule out the possibility of unmeasured confounding. Because WBC count is a sensitive hematologic marker for various physical and psychosocial stressors, future studies may need to consider other contextual features (e.g., crime rate, characteristics of built environment) of the residential environment that are associated with CVD risks and may also covary with ambient air pollution levels. Third, using historical environmental monitoring data as the only information source for an individual-level exposure assignment, we were unable to relate the spatial difference in WBC count to other related features of ambient PM exposure beyond the varying PM_10_ levels across the communities. For instance, we did not know whether there were similar associations with long-term exposure to ambient PM of different size characterization (e.g., PM_2.5_, coarse PM). Although the effects of other copollutants were not the focus of the present study, we did conduct some additional analyses and did not find associations between WBC count and estimated levels of other ambient air pollutants, including O_3_, NO_2_, and SO_2_ (data not shown). Fourth, because no nationwide PM speciation data were available for 1988–1991, we failed to identify specific constituents or investigate any important PM_10_ sources responsible for the putative long-term inflammatory responses to ambient air particles. Given that so much of the observed exposure variability (94%) is dominated by PM_10_ gradients across communities, our study findings may imply some inflammatory responses induced by exposure to long-term PM of regional sources. Although earlier cohort analyses on cardiopulmonary mortality spoke to the importance of regional-scale PM sources, more recent studies were able to show the effect on CVD across PM gradients within cities (Jerrett et al. 2005; Miller et al. 2007), suggesting the significance of local PM sources as well. Whether long-term exposure to PM of local-scale sources can induce inflammatory responses needs further investigation. Fifth, because most PM_10_ data were monitored only every 6 days, which rarely coincided with the NHANES III examination day, there were only 708 subjects within our defined study population (*n* = 2,978) for whom both daily and 1-year PM_10_ data were available concurrently with the WBC measurement, making it impractical to jointly model the presumably independent effects of daily and annual average PM_10_ exposures. Nonetheless, we noted in our restricted sample that the correlation coefficient between daily and 1-year PM_10_ estimates was 0.09. This empirically modest correlation suggests that short-term PM exposure contributes only a small portion, if any, of the overall inflammatory response to long-term repeated PM exposure. Finally, increased WBC count is a nonspecific hematologic marker of inflammation. Future epidemiologic data on more specific molecular markers (e.g., expression of C-reactive protein or other biomarkers of vascular inflammation) are needed to confirm previous animal and *in vitro* studies.

## Conclusions

The consistent PM_10_-related spatial difference in WBC count in our study denotes the presence of inflammatory responses involved in either the development or progression of CVD associated with long-term PM exposure. The observed increasing inflammatory response across subpopulations with more MS component abnormalities supports the concept of MS-dependent susceptibility to PM-associated long-term cardiovascular effects.

## Figures and Tables

**Table 1:** Demographic characteristics of current study participants and source population in the first period of NHANES III, 1988–1991.

Characteristic	Adult subjects with missing PM_10_ data	Adults subjects with estimable PM_10_ data	Current study population
Age (years)	3,037 (49.6 ± 20.4)	5,369 (48.9 ± 19.3)	2,978 (48.5 ± 17.8)
Ethnicity			
Non-Hispanic white	1,728 (57)	1,995 (37)	1,176 (39)
Non-Hispanic black	749 (25)	1,415 (26)	678 (23)
Mexican American	530 (18)	1,726 (32)	1,008 (39)
Others	30 (1)	233 (4)	116 (4)
Sex			
Male	1,401 (49)	2,716 (51)	1,527 (51)
Female	1,461 (51)	2,653 (49)	1,451 (49)
Years of education	3,014 (10.7 ± 3.7)	5,328 (11.0 ± 4.1)	2,962 (11.1 ± 5.1)
Annual family income			
< $20,000	1,403 (52)	2,184 (46)	1,085 (41)
$20,000–35,000	711 (26)	1,160 (24)	660 (25)
$35,000–50,000	330 (12)	664 (14)	430 (16)
> $50,000	266 (10)	748 (16)	494 (18)
Poverty–income ratio	2,561 (2.07 ± 1.48)	4,757 (2.46 ± 1.64)	2,669 (2.62 ± 1.67)
Urbanization			
Urban	539 (18)	3,622 (67)	2,054 (69)
Rural	2,498 (82)	1,747 (33)	924 (31)
Smoking			
Never	1,348 (44)	2,542 (48)	1,417 (48)
Past	775 (26)	1,423 (27)	829 (28)
Active	914 (30)	1,404 (26)	732 (25)
MS			
No	1,718 (71.4)	3,435 (74.8)	2,229 (74.9)
Yes	689 (28.6)	1,158 (25.2)	749 (25.1)
WBC count (× 10^6^/L)	2,651 (7,309 ± 2,370)	4,975 (7,142 ± 2,263)	2,978 (6,909 ± 1,617)

Values are no. (%) or no. (mean ± SD). The total number of subjects summed across each subcategory varies slightly because of missing values.

**Table 2:** Population correlates of quartile distribution of estimated 1-year average PM_10_ exposure.

Characteristic	1st	2nd	3rd	4th
Age (years)	835 (50.9 ± 18.4)	687 (49.9 ± 17.6)	726 (46.9 ± 17.8)	730 (46.0 ± 16.8)
Ethnicity			
Non-Hispanic white	434 (52)	297 (43)	293 (40)	152 (21)
Non-Hispanic black	226 (27)	133 (19)	237 (33)	82 (11)
Hispanics and others	175 (21)	257 (37)	196 (27)	496 (68)
Sex			
Male	422 (51)	368 (54)	368 (51)	369 (5)
Female	413 (49)	319 (46)	358 (49)	361 (49)
Years of education	832 (11.7 ± 3.8)	685 (11.4 ± 4.2)	721 (11.2 ± 3.7)	724 (10.1 ± 4.7)
Annual family income > $50,000			
No	561 (75)	503 (80)	554 (85)	557 (88)
Yes	187 (25)	129 (20)	101 (15)	77 (12)
Poverty–income ratio	748 (2.87 ± 1.76)	632 (2.82 ± 1.76)	655 (2.35 ± 1.52)	634 (2.42 ± 1.55)
Urbanization			
Urban	444 (53)	486 (71)	560 (77)	564 (77)
Rural	391 (47)	201 (29)	166 (23)	166 (23)
Smoking			
Never	399 (48)	320 (47)	313 (43)	385 (53)
Past	247 (30)	206 (30)	195 (27)	181 (25)
Active	189 (23)	161 (23)	218 (30)	164 (23)
Metabolic syndrome			
No	61 (73.9)	520 (75.7)	567 (78.1)	525 (71.9)
Yes	218 (26.1)	167 (24.3)	159 (21.9)	205 (28.1)

Values are no. (%) or no. (mean ± SD). Total number of subjects summed across each subcategory varies slightly because of missing values.

**Table 3:** Spatial difference in average WBC count across communities with different levels of estimated 1-year average PM_10_ exposure, by quartile.

	1st	2nd	3rd	4th
Average WBC count ± SE^a^ (× 10^6^/L)	6,760 ± 79	6,942 ± 99	6,895 ± 84	7,109 ± 61
Median (range) of 1-year PM_10_ (μg/m^3^)	23.1 (14.6–27.8)	31.2 (27.9–34.3)	38.8 (34.3–43.3)	53.7 (43.3–78.5)

a Average WBC count and SE estimated by mixed-effects modeling adjusted for within-cluster correlation.

**Table 4:** Mixed-effects modeling of spatial difference in WBC count associated with 1-year average PM_10_ exposure.

	Crude	Model 1^a^	Model 2^b^	Model 3^c^
β (× 10^6^/L)^d^	239	145	141	138
95% CI^d^	58–420	10–281	6–277	2–273
*p*-Value	0.01	0.035	0.041	0.046

a Adjusted for within-cluster correlation, age, sex, race, socioeconomic factors (education, income, employment status, poverty–income ratio, family size), smoking status, alcohol consumption, urban/rural designation, and number of MS abnormalities.

b Model 1 plus additional adjustment for sources of indoor air pollutants (environmental tobacco smoke, wood stove, fireplace, gas stove).

c Model 1 plus additional adjustment for indoor air pollutants (environmental tobacco smoke, wood stove, fireplace, gas stove) and exercise activities (jogging/running, aerobic exercise).

d Regression coefficient and 95% CI representing the difference in average WBC count comparing participants residing in clean air communities (1st quartile of 1-year PM_10_) with those living in other, more polluted areas.
